# 13-(Imidazol-1-yl)-11,13-di­hydro­melampomagnolide B monohydrate

**DOI:** 10.1107/S1600536813029188

**Published:** 2013-11-06

**Authors:** Venumadhav Janganati, Narsimha Reddy Penthala, Sean Parkin, Peter A. Crooks

**Affiliations:** a Department of Pharmaceutical Sciences, College of Pharmacy, University of Arkansas for Medical Sciences, Little Rock, AR 72205, USA; b Department of Chemistry, University of Kentucky, Lexington KY 40506, USA

## Abstract

The title compound, C_18_H_24_N_2_O_4_·H_2_O {systematic name: (1a*R*,7a*S*,8*R*,10a*S*,10b*S*,*E*)-5-hy­droxy­methyl-8-[(1*H*-imidazol-1-yl)meth­yl]-1a-methyl-2,3,6,7,7a,8,10a,10b-octa­hydro­oxireno[2′,3′:9,10]cyclo­deca­[1,2-b]furan-9(1a*H*)-one monohydrate}, an imidazole derivative of melampomagnolide B was synthesized under Michael addition conditions. The mol­ecule is built up from fused ten-, five- (lactone) and three-membered (epoxide) rings. The inter­nal double bond of the ten-membered ring identifies it as the *cis* or *E* isomer. The lactone ring has an envelope-type conformation, with the (chiral) C atom opposite the lactone O atoms as the flap atom. In the crystal, O—H⋯O, O—H⋯N and weak C—H⋯O hydrogen bonds link the mol­ecules (along with water) into sheets parallel to the *bc* plane.

## Related literature

For the biological activity of similar compounds, see: El-Feraly (1984 ▶); Macias *et al.* (1992 ▶); Nasim *et al.* (2011 ▶); Nasim & Crooks (2008 ▶). For the structures of similar compounds, see; Neelakantan *et al.* (2009 ▶); Woods *et al.* (2011 ▶); Neukirch *et al.* (2003 ▶); Gonzalez *et al.* (1988 ▶).
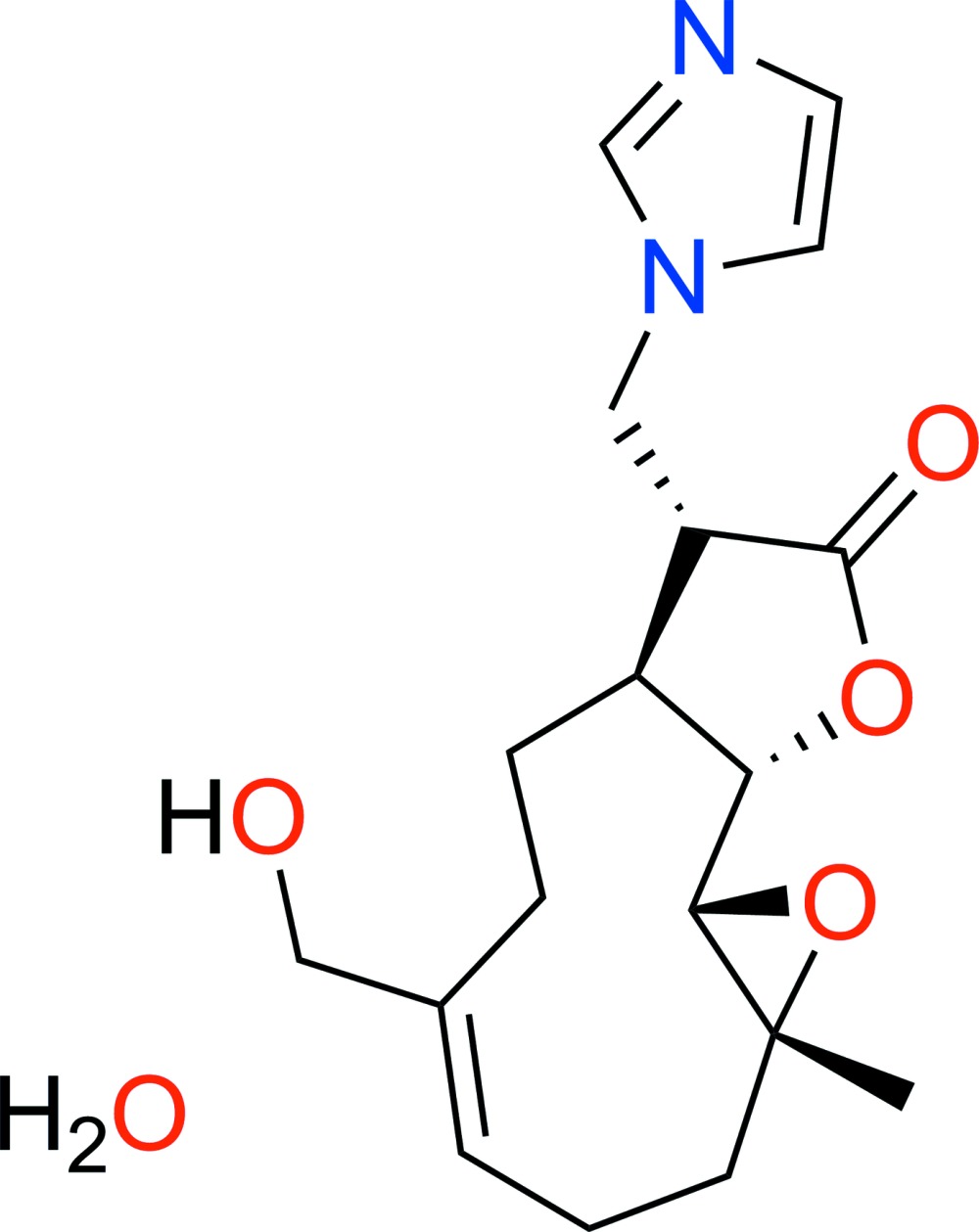


## Experimental

### 

#### Crystal data

C_18_H_24_N_2_O_4_·H_2_O
*M* *_r_* = 350.41
Monoclinic, 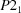

*a* = 9.8073 (2) Å
*b* = 8.2784 (1) Å
*c* = 10.7741 (2) Å
β = 95.015 (1)°
*V* = 871.39 (3) Å^3^
*Z* = 2
Cu *K*α radiation
μ = 0.80 mm^−1^
*T* = 90 K
0.25 × 0.20 × 0.04 mm

#### Data collection

Bruker X8 Proteum diffractometer
Absorption correction: multi-scan (*SADABS*; Sheldrick, 2008*a* ▶) *T*_min_ = 0.836, *T*_max_ = 0.942
10767 measured reflections
2437 independent reflections
2425 reflections with *I* > 2σ(*I*)
*R*_int_ = 0.030

#### Refinement

*R*[*F* ^2^ > 2σ(*F* ^2^)] = 0.024
*wR*(*F* ^2^) = 0.061
*S* = 1.04
2437 reflections
235 parameters
1 restraint
H atoms treated by a mixture of independent and constrained refinement
Δρ_max_ = 0.16 e Å^−3^
Δρ_min_ = −0.15 e Å^−3^
Absolute structure: Flack parameter determined using 747 quotients [(*I* ^+^)−(*I* ^−^)]/[(*I* ^+^)+(*I* ^−^)] (Parsons *et al.*, 2013 ▶)
Absolute structure parameter: −0.03 (5)

### 

Data collection: *APEX2* (Bruker, 2006 ▶); cell refinement: *SAINT* (Bruker, 2006 ▶); data reduction: *SAINT*; program(s) used to solve structure: *SHELXS97* (Sheldrick, 2008*b* ▶); program(s) used to refine structure: *SHELXL2013* (Sheldrick, 2008*b* ▶); molecular graphics: *XP* in *SHELXTL* (Sheldrick, 2008*b* ▶); software used to prepare material for publication: *SHELXL2013*.

## Supplementary Material

Crystal structure: contains datablock(s) global, I. DOI: 10.1107/S1600536813029188/sj5362sup1.cif

Structure factors: contains datablock(s) I. DOI: 10.1107/S1600536813029188/sj5362Isup2.hkl

Additional supplementary materials:  crystallographic information; 3D view; checkCIF report

## Figures and Tables

**Table 1:** Hydrogen-bond geometry (Å, °)

*D*—H⋯*A*	*D*—H	H⋯*A*	*D*⋯*A*	*D*—H⋯*A*
O4—H4⋯N2^i^	0.84	1.93	2.7491 (17)	164
C13—H13*B*⋯O1*W* ^ii^	0.99	2.45	3.388 (2)	157
C15—H15*B*⋯O3^iii^	0.98	2.55	3.282 (2)	131
C16—H16*A*⋯O1*W* ^iv^	0.95	2.44	3.370 (2)	167
C17—H17*A*⋯O3^v^	0.95	2.54	3.377 (2)	147
C18—H18*A*⋯O1^v^	0.95	2.57	3.5016 (19)	167
O1*W*—H1*W*⋯O4	0.87 (3)	1.93 (3)	2.7928 (17)	173 (3)
O1*W*—H2*W*⋯O3^vi^	0.85 (3)	2.14 (3)	2.9534 (19)	162 (2)

Symmetry codes: (i) 
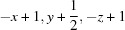
; (ii) 
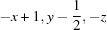
; (iii) 
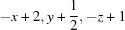
; (iv) 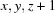; (v) 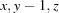; (vi) 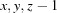.

**Fig. 1.**
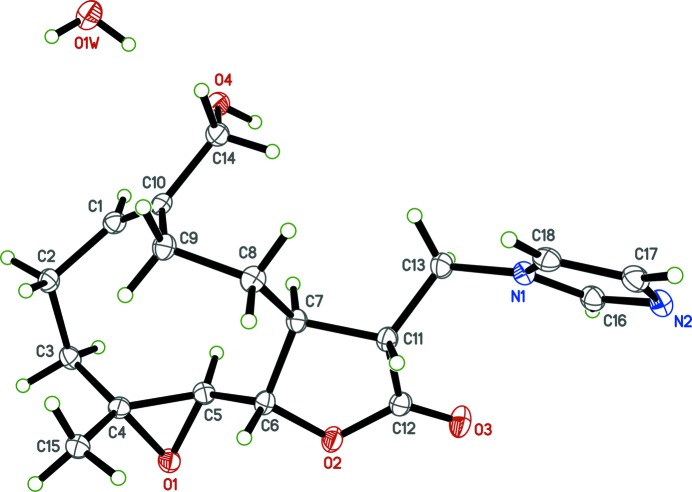
The title molecule with the atom numbering scheme. Displacement ellipsoids are drawn at the 50% probability level.

## supplementary crystallographic information

### 1. Comment

Melampomagnolide B (MMB) was derived from parthenolide (PTL) via selenium oxide mediated oxidation of the C10 methyl group of PTL (Macias *et al.*, 1992; Neukirch *et al.*, 2003). MMB is a melampolide originally isolated from *Magnolia grandiflora* and characterized by X-ray diffraction analysis (El-Feraly, 1984; Gonzalez *et al.*, 1988), and has been identified as a new anti-leukemic sesquiterpene with properties similar to PTL (Nasim *et al.*, 2011).

Recently, Nasim *et al.* (2008) reported formation of amino-parthenolide derivatives under Michael addition reaction conditions. These compounds showed more potency as antileukemic agents and improved water solubility relative to PTL. To further improve water solubility of melampomagnolide B, we synthesized an imidazole derivative by the reaction of MMB with imidazole via Michael addition chemistry (Neelakantan *et al.*, 2009; Woods *et al.*, 2011) to afford the title compound, which was re-crystallized from chloroform.

To obtain detailed information on the structural conformation of this molecule, establish the geometry of the double bond (C9═C10) and orientation of the imidazole moiety, a single-crystal X-ray structure determination has been carried out. This has revealed that the double bond of the title compound has the *E*-geometry. In the crystal, intermolecular O—H···O, O—H···N and weak C—H···O hydrogen bonds link the molecules (along with water) into sheets parallel to the *bc*-plane.

### 2. Experimental

To a solution of MMB (50 mg, 0.189 m mol) in methanol, was added imidazole (19.31 mg, 0.284 m mol). The reaction mixture was stirred at ambient temperature for 24 h. After completion of the reaction (monitored by thin-layer chromatography), the reaction mixture was dissolved in dichloromethane. The organic layer was separated, washed with water, dried over anhydrous Na_2_SO_4_ and concentrated under reduced pressure to afford the crude product. The crude compound was recrystallized from chloroform to obtain the title compound as colorless crystals (56 mg, yield: 89%). Melting point 398-399°K. ^1^H NMR (400 MHz, CDCl_3_): δ 7.60 (s, 1H), 7.10 (s, 1H), 7.01 (s, 1H), 5.55 (t, *J*=8Hz, 1H), 4.48 (dd, *J*=14.4 Hz, 1H), 4.32 (dd, *J*=14.8, 1H),3.93 (s, 2H), 3.89(t, *J*=9.6Hz, 1H), 2.68 (d, *J*=9.2 Hz, 2H), 2.34-1.90 (m, 11H), 1.88-1.53 (m, 3H), 1.04 (t, *J*=9.6 Hz, 1H). ^13^C NMR (CDCl_3_, 100 MHz): δ 174.9, 139.1, 138.0, 130.3, 126.8, 119.4, 81.2, 65.2, 62.5, 60.3, 48.4, 43.8, 41.6, 36.7, 26.9, 24.6, 23.4, 17.9 *ppm*.

### 3. Refinement

H atoms were found in difference Fourier maps and subsequently placed at idealized positions with constrained distances of 0.98 Å (RCH_3_), 0.99 Å (R_2_CH_2_), 1.00 Å (R_3_CH), 0.95 Å (C_sp2_H). Water hydrogen coordinates were refined. U_iso_(H) values were set to either 1.2U_eq_ or 1.5U_eq_ (RCH_3_, OH) of the attached atom.

### Crystal data

**Table:**

C_18_H_24_N_2_O_4_·H_2_O	*F*(000) = 376
*M**_r_* = 350.41	*D*_x_ = 1.335 Mg m^−^^3^
Monoclinic, *P*2_1_	Cu *K*α radiation, λ = 1.54178 Å
*a* = 9.8073 (2) Å	Cell parameters from 9966 reflections
*b* = 8.2784 (1) Å	θ = 4.1–68.1°
*c* = 10.7741 (2) Å	µ = 0.80 mm^−^^1^
β = 95.015 (1)°	*T* = 90 K
*V* = 871.39 (3) Å^3^	Tablet, colourless
*Z* = 2	0.25 × 0.20 × 0.04 mm

### Data collection

**Table:**

Bruker X8 Proteum diffractometer	2437 independent reflections
Radiation source: fine-focus rotating anode	2425 reflections with *I* > 2σ(*I*)
Detector resolution: 5.6 pixels mm^-1^	*R*_int_ = 0.030
φ and ω scans	θ_max_ = 68.1°, θ_min_ = 4.5°
Absorption correction: multi-scan (*SADABS*; Sheldrick, 2008*a*)	*h* = −11→11
*T*_min_ = 0.836, *T*_max_ = 0.942	*k* = −9→7
10767 measured reflections	*l* = −12→11

### Refinement

**Table:**

Refinement on *F*^2^	Hydrogen site location: difference Fourier map
Least-squares matrix: full	H atoms treated by a mixture of independent and constrained refinement
*R*[*F*^2^ > 2σ(*F*^2^)] = 0.024	*w* = 1/[σ^2^(*F*_o_^2^) + (0.0332*P*)^2^ + 0.170*P*] where *P* = (*F*_o_^2^ + 2*F*_c_^2^)/3
*wR*(*F*^2^) = 0.061	(Δ/σ)_max_ < 0.001
*S* = 1.04	Δρ_max_ = 0.16 e Å^−^^3^
2437 reflections	Δρ_min_ = −0.15 e Å^−^^3^
235 parameters	Extinction correction: *SHELXL2013* (Sheldrick, 2008a), Fc^*^=kFc[1+0.001xFc^2^λ^3^/sin(2θ)]^-1/4^
1 restraint	Extinction coefficient: 0.0090 (15)
Primary atom site location: structure-invariant direct methods	Absolute structure: Flack parameter determined using 747 quotients [(*I*^+^)-(*I*^-^)]/[(*I*^+^)+(*I*^-^)] (Parsons *et al.*, 2013)
Secondary atom site location: difference Fourier map	Absolute structure parameter: −0.03 (5)

### Special details

**Table:**

Geometry. All esds (except the esd in the dihedral angle between two l.s. planes) are estimated using the full covariance matrix. The cell esds are taken into account individually in the estimation of esds in distances, angles and torsion angles; correlations between esds in cell parameters are only used when they are defined by crystal symmetry. An approximate (isotropic) treatment of cell esds is used for estimating esds involving l.s. planes.

### Fractional atomic coordinates and isotropic or equivalent isotropic displacement parameters (Å^2^)

**Table:**

	*x*	*y*	*z*	*U*_iso_*/*U*_eq_	
N1	0.63838 (13)	0.11358 (19)	0.56147 (12)	0.0159 (3)	
N2	0.61292 (14)	−0.0075 (2)	0.74068 (12)	0.0197 (3)	
O1	0.92738 (12)	0.85603 (16)	0.33577 (10)	0.0183 (3)	
O2	0.83645 (12)	0.60393 (16)	0.51264 (9)	0.0185 (3)	
O3	0.71648 (13)	0.48872 (17)	0.65518 (10)	0.0221 (3)	
O4	0.47506 (11)	0.43708 (16)	0.02773 (10)	0.0179 (3)	
H4	0.4364	0.4648	0.0910	0.027*	
C1	0.71890 (15)	0.6089 (2)	0.02275 (13)	0.0161 (3)	
H1A	0.6293	0.6497	0.0017	0.019*	
C2	0.83497 (16)	0.7229 (2)	0.00040 (14)	0.0163 (3)	
H2A	0.8112	0.7803	−0.0791	0.020*	
H2B	0.9178	0.6576	−0.0099	0.020*	
C3	0.87106 (16)	0.8502 (2)	0.10296 (14)	0.0166 (4)	
H3A	0.9320	0.9330	0.0713	0.020*	
H3B	0.7864	0.9046	0.1245	0.020*	
C4	0.94124 (16)	0.7722 (2)	0.21808 (14)	0.0153 (3)	
C5	0.85016 (16)	0.7092 (2)	0.30889 (14)	0.0152 (3)	
H5A	0.7506	0.7274	0.2847	0.018*	
C6	0.87955 (16)	0.5643 (2)	0.38885 (14)	0.0152 (4)	
H6A	0.9790	0.5362	0.3940	0.018*	
C7	0.79086 (15)	0.4188 (2)	0.34526 (13)	0.0141 (3)	
H7A	0.7011	0.4603	0.3069	0.017*	
C8	0.85003 (16)	0.3049 (2)	0.25191 (14)	0.0165 (4)	
H8A	0.7947	0.2047	0.2465	0.020*	
H8B	0.9442	0.2750	0.2844	0.020*	
C9	0.85478 (15)	0.3742 (2)	0.11888 (14)	0.0164 (4)	
H9A	0.9322	0.4511	0.1193	0.020*	
H9B	0.8734	0.2847	0.0618	0.020*	
C10	0.72548 (15)	0.4597 (2)	0.06786 (13)	0.0147 (3)	
C11	0.76625 (16)	0.3373 (2)	0.46888 (14)	0.0153 (3)	
H11A	0.8457	0.2653	0.4942	0.018*	
C12	0.76819 (16)	0.4785 (2)	0.55787 (14)	0.0168 (3)	
C13	0.63503 (16)	0.2376 (2)	0.46427 (14)	0.0179 (4)	
H13A	0.5567	0.3107	0.4735	0.021*	
H13B	0.6201	0.1848	0.3817	0.021*	
C14	0.59851 (15)	0.3551 (2)	0.06538 (14)	0.0168 (4)	
H14A	0.6089	0.2634	0.0080	0.020*	
H14B	0.5918	0.3099	0.1497	0.020*	
C15	1.08469 (16)	0.7115 (2)	0.20694 (15)	0.0181 (4)	
H15A	1.1182	0.6563	0.2841	0.027*	
H15B	1.1448	0.8029	0.1925	0.027*	
H15C	1.0843	0.6358	0.1370	0.027*	
C16	0.58471 (15)	0.1233 (2)	0.67287 (14)	0.0178 (4)	
H16A	0.5334	0.2126	0.6989	0.021*	
C17	0.68861 (16)	−0.1053 (2)	0.66946 (15)	0.0186 (4)	
H17A	0.7239	−0.2084	0.6940	0.022*	
C18	0.70535 (15)	−0.0320 (2)	0.55829 (14)	0.0167 (3)	
H18A	0.7534	−0.0731	0.4923	0.020*	
O1W	0.45357 (13)	0.47330 (19)	−0.23096 (12)	0.0248 (3)	
H1W	0.458 (2)	0.470 (4)	−0.150 (2)	0.037*	
H2W	0.534 (3)	0.492 (4)	−0.249 (2)	0.037*	

### Atomic displacement parameters (Å^2^)

**Table:**

	*U*^11^	*U*^22^	*U*^33^	*U*^12^	*U*^13^	*U*^23^
N1	0.0161 (6)	0.0167 (8)	0.0149 (6)	−0.0029 (6)	0.0018 (5)	0.0004 (6)
N2	0.0210 (7)	0.0198 (8)	0.0187 (6)	−0.0055 (6)	0.0036 (5)	0.0015 (6)
O1	0.0228 (5)	0.0157 (7)	0.0168 (5)	−0.0042 (5)	0.0042 (4)	−0.0020 (5)
O2	0.0261 (6)	0.0167 (7)	0.0131 (5)	−0.0028 (5)	0.0043 (4)	−0.0003 (5)
O3	0.0317 (6)	0.0194 (7)	0.0163 (5)	0.0021 (6)	0.0088 (5)	0.0018 (5)
O4	0.0141 (5)	0.0227 (7)	0.0170 (5)	0.0000 (5)	0.0021 (4)	−0.0003 (5)
C1	0.0143 (7)	0.0191 (9)	0.0149 (7)	0.0006 (7)	0.0003 (5)	−0.0004 (7)
C2	0.0173 (7)	0.0167 (9)	0.0147 (7)	0.0001 (7)	0.0005 (5)	0.0041 (7)
C3	0.0163 (7)	0.0151 (9)	0.0186 (7)	−0.0016 (7)	0.0022 (6)	0.0021 (7)
C4	0.0175 (7)	0.0134 (9)	0.0150 (7)	−0.0018 (6)	0.0014 (6)	−0.0012 (6)
C5	0.0161 (7)	0.0138 (9)	0.0157 (7)	0.0000 (7)	0.0022 (6)	−0.0008 (7)
C6	0.0161 (7)	0.0168 (10)	0.0130 (7)	0.0004 (6)	0.0031 (6)	0.0003 (6)
C7	0.0157 (7)	0.0130 (9)	0.0139 (7)	0.0004 (7)	0.0019 (5)	0.0019 (6)
C8	0.0185 (7)	0.0144 (9)	0.0166 (7)	0.0023 (7)	0.0016 (6)	0.0006 (7)
C9	0.0170 (7)	0.0172 (9)	0.0154 (7)	0.0014 (7)	0.0038 (6)	−0.0005 (7)
C10	0.0160 (7)	0.0174 (9)	0.0109 (6)	−0.0005 (7)	0.0021 (5)	−0.0018 (6)
C11	0.0171 (7)	0.0152 (9)	0.0135 (7)	0.0000 (7)	0.0012 (5)	0.0023 (6)
C12	0.0186 (7)	0.0159 (9)	0.0156 (7)	0.0002 (7)	−0.0008 (6)	0.0030 (6)
C13	0.0182 (7)	0.0188 (10)	0.0165 (7)	−0.0015 (7)	0.0001 (6)	0.0045 (7)
C14	0.0184 (7)	0.0157 (9)	0.0164 (7)	−0.0006 (7)	0.0019 (5)	0.0001 (7)
C15	0.0152 (7)	0.0223 (10)	0.0168 (7)	−0.0015 (7)	0.0006 (6)	0.0013 (7)
C16	0.0162 (7)	0.0190 (10)	0.0187 (7)	−0.0020 (7)	0.0044 (6)	−0.0017 (7)
C17	0.0194 (7)	0.0144 (9)	0.0216 (8)	−0.0017 (7)	−0.0005 (6)	0.0009 (7)
C18	0.0174 (7)	0.0141 (9)	0.0186 (7)	−0.0012 (6)	0.0014 (6)	−0.0039 (7)
O1W	0.0252 (6)	0.0304 (8)	0.0190 (6)	0.0031 (6)	0.0024 (5)	0.0011 (6)

### Geometric parameters (Å, º)

**Table:**

N1—C16	1.354 (2)	C7—C8	1.529 (2)
N1—C18	1.374 (2)	C7—C11	1.531 (2)
N1—C13	1.465 (2)	C7—H7A	1.0000
N2—C16	1.322 (2)	C8—C9	1.549 (2)
N2—C17	1.377 (2)	C8—H8A	0.9900
O1—C5	1.448 (2)	C8—H8B	0.9900
O1—C4	1.4623 (19)	C9—C10	1.513 (2)
O2—C12	1.350 (2)	C9—H9A	0.9900
O2—C6	1.4709 (18)	C9—H9B	0.9900
O3—C12	1.207 (2)	C10—C14	1.515 (2)
O4—C14	1.416 (2)	C11—C12	1.511 (2)
O4—H4	0.8400	C11—C13	1.526 (2)
C1—C10	1.327 (3)	C11—H11A	1.0000
C1—C2	1.514 (2)	C13—H13A	0.9900
C1—H1A	0.9500	C13—H13B	0.9900
C2—C3	1.545 (2)	C14—H14A	0.9900
C2—H2A	0.9900	C14—H14B	0.9900
C2—H2B	0.9900	C15—H15A	0.9800
C3—C4	1.510 (2)	C15—H15B	0.9800
C3—H3A	0.9900	C15—H15C	0.9800
C3—H3B	0.9900	C16—H16A	0.9500
C4—C5	1.477 (2)	C17—C18	1.365 (2)
C4—C15	1.509 (2)	C17—H17A	0.9500
C5—C6	1.490 (2)	C18—H18A	0.9500
C5—H5A	1.0000	O1W—H1W	0.87 (3)
C6—C7	1.536 (2)	O1W—H2W	0.85 (3)
C6—H6A	1.0000		
			
C16—N1—C18	107.33 (15)	C7—C8—H8B	108.5
C16—N1—C13	127.27 (16)	C9—C8—H8B	108.5
C18—N1—C13	125.28 (14)	H8A—C8—H8B	107.5
C16—N2—C17	105.71 (14)	C10—C9—C8	114.69 (13)
C5—O1—C4	60.97 (10)	C10—C9—H9A	108.6
C12—O2—C6	110.30 (13)	C8—C9—H9A	108.6
C14—O4—H4	109.5	C10—C9—H9B	108.6
C10—C1—C2	128.70 (15)	C8—C9—H9B	108.6
C10—C1—H1A	115.6	H9A—C9—H9B	107.6
C2—C1—H1A	115.7	C1—C10—C9	125.51 (15)
C1—C2—C3	116.07 (13)	C1—C10—C14	120.80 (14)
C1—C2—H2A	108.3	C9—C10—C14	113.61 (15)
C3—C2—H2A	108.3	C12—C11—C13	113.76 (13)
C1—C2—H2B	108.3	C12—C11—C7	102.53 (14)
C3—C2—H2B	108.3	C13—C11—C7	113.99 (12)
H2A—C2—H2B	107.4	C12—C11—H11A	108.8
C4—C3—C2	110.81 (14)	C13—C11—H11A	108.8
C4—C3—H3A	109.5	C7—C11—H11A	108.8
C2—C3—H3A	109.5	O3—C12—O2	121.27 (17)
C4—C3—H3B	109.5	O3—C12—C11	128.51 (17)
C2—C3—H3B	109.5	O2—C12—C11	110.21 (13)
H3A—C3—H3B	108.1	N1—C13—C11	112.93 (12)
O1—C4—C5	59.05 (10)	N1—C13—H13A	109.0
O1—C4—C15	112.65 (12)	C11—C13—H13A	109.0
C5—C4—C15	123.88 (15)	N1—C13—H13B	109.0
O1—C4—C3	116.05 (14)	C11—C13—H13B	109.0
C5—C4—C3	115.89 (13)	H13A—C13—H13B	107.8
C15—C4—C3	116.01 (13)	O4—C14—C10	114.33 (14)
O1—C5—C4	59.98 (10)	O4—C14—H14A	108.7
O1—C5—C6	119.25 (12)	C10—C14—H14A	108.7
C4—C5—C6	124.74 (15)	O4—C14—H14B	108.7
O1—C5—H5A	114.0	C10—C14—H14B	108.7
C4—C5—H5A	114.0	H14A—C14—H14B	107.6
C6—C5—H5A	114.0	C4—C15—H15A	109.5
O2—C6—C5	106.78 (14)	C4—C15—H15B	109.5
O2—C6—C7	104.61 (12)	H15A—C15—H15B	109.5
C5—C6—C7	112.27 (12)	C4—C15—H15C	109.5
O2—C6—H6A	111.0	H15A—C15—H15C	109.5
C5—C6—H6A	111.0	H15B—C15—H15C	109.5
C7—C6—H6A	111.0	N2—C16—N1	111.24 (16)
C8—C7—C11	113.47 (14)	N2—C16—H16A	124.4
C8—C7—C6	116.59 (13)	N1—C16—H16A	124.4
C11—C7—C6	102.00 (12)	C18—C17—N2	109.84 (16)
C8—C7—H7A	108.1	C18—C17—H17A	125.1
C11—C7—H7A	108.1	N2—C17—H17A	125.1
C6—C7—H7A	108.1	C17—C18—N1	105.87 (14)
C7—C8—C9	115.08 (14)	C17—C18—H18A	127.1
C7—C8—H8A	108.5	N1—C18—H18A	127.1
C9—C8—H8A	108.5	H1W—O1W—H2W	106 (2)
			
C10—C1—C2—C3	−98.0 (2)	C2—C1—C10—C14	−170.69 (14)
C1—C2—C3—C4	72.09 (17)	C8—C9—C10—C1	126.24 (17)
C5—O1—C4—C15	−117.00 (17)	C8—C9—C10—C14	−56.95 (19)
C5—O1—C4—C3	105.88 (16)	C8—C7—C11—C12	156.85 (12)
C2—C3—C4—O1	−154.00 (13)	C6—C7—C11—C12	30.61 (15)
C2—C3—C4—C5	−87.53 (18)	C8—C7—C11—C13	−79.73 (17)
C2—C3—C4—C15	70.32 (19)	C6—C7—C11—C13	154.03 (14)
C4—O1—C5—C6	115.52 (17)	C6—O2—C12—O3	−175.49 (14)
C15—C4—C5—O1	97.94 (17)	C6—O2—C12—C11	3.08 (17)
C3—C4—C5—O1	−106.14 (16)	C13—C11—C12—O3	32.8 (2)
O1—C4—C5—C6	−106.62 (16)	C7—C11—C12—O3	156.40 (16)
C15—C4—C5—C6	−8.7 (3)	C13—C11—C12—O2	−145.62 (13)
C3—C4—C5—C6	147.24 (15)	C7—C11—C12—O2	−22.05 (16)
C12—O2—C6—C5	136.46 (13)	C16—N1—C13—C11	97.44 (18)
C12—O2—C6—C7	17.28 (15)	C18—N1—C13—C11	−78.22 (19)
O1—C5—C6—O2	67.27 (17)	C12—C11—C13—N1	−85.46 (18)
C4—C5—C6—O2	139.25 (15)	C7—C11—C13—N1	157.44 (14)
O1—C5—C6—C7	−178.64 (13)	C1—C10—C14—O4	−8.0 (2)
C4—C5—C6—C7	−106.66 (17)	C9—C10—C14—O4	174.99 (12)
O2—C6—C7—C8	−153.79 (12)	C17—N2—C16—N1	0.21 (18)
C5—C6—C7—C8	90.80 (17)	C18—N1—C16—N2	−0.26 (18)
O2—C6—C7—C11	−29.62 (15)	C13—N1—C16—N2	−176.55 (14)
C5—C6—C7—C11	−145.03 (13)	C16—N2—C17—C18	−0.07 (17)
C11—C7—C8—C9	170.63 (12)	N2—C17—C18—N1	−0.08 (17)
C6—C7—C8—C9	−71.29 (17)	C16—N1—C18—C17	0.20 (17)
C7—C8—C9—C10	−45.1 (2)	C13—N1—C18—C17	176.58 (13)
C2—C1—C10—C9	5.9 (3)		

### Hydrogen-bond geometry (Å, º)

**Table:**

*D*—H···*A*	*D*—H	H···*A*	*D*···*A*	*D*—H···*A*
O4—H4···N2^i^	0.84	1.93	2.7491 (17)	164
C13—H13*B*···O1*W*^ii^	0.99	2.45	3.388 (2)	157
C15—H15*B*···O3^iii^	0.98	2.55	3.282 (2)	131
C16—H16*A*···O1*W*^iv^	0.95	2.44	3.370 (2)	167
C17—H17*A*···O3^v^	0.95	2.54	3.377 (2)	147
C18—H18*A*···O1^v^	0.95	2.57	3.5016 (19)	167
O1*W*—H1*W*···O4	0.87 (3)	1.93 (3)	2.7928 (17)	173 (3)
O1*W*—H2*W*···O3^vi^	0.85 (3)	2.14 (3)	2.9534 (19)	162 (2)

Symmetry codes: (i) −*x*+1, *y*+1/2, −*z*+1; (ii) −*x*+1, *y*−1/2, −*z*; (iii) −*x*+2, *y*+1/2, −*z*+1; (iv) *x*, *y*, *z*+1; (v) *x*, *y*−1, *z*; (vi) *x*, *y*, *z*−1.
